# The Identification of Markers of Macrophage Differentiation in PMA-Stimulated THP-1 Cells and Monocyte-Derived Macrophages

**DOI:** 10.1371/journal.pone.0008668

**Published:** 2010-01-13

**Authors:** Marc Daigneault, Julie A. Preston, Helen M. Marriott, Moira K. B. Whyte, David H. Dockrell

**Affiliations:** Department of Infection and Immunity, Medical School, University of Sheffield, Sheffield, United Kingdom; Statens Serum Institute, Denmark

## Abstract

Differentiated macrophages are the resident tissue phagocytes and sentinel cells of the innate immune response. The phenotype of mature tissue macrophages represents the composite of environmental and differentiation-dependent imprinting. Phorbol-12-myristate-13-acetate (PMA) and 1,25-dihydroxyvitamin D3 (VD_3_) are stimuli commonly used to induce macrophage differentiation in monocytic cell lines but the extent of differentiation in comparison to primary tissue macrophages is unclear. We have compared the phenotype of the promonocytic THP-1 cell line after various protocols of differentiation utilising VD_3_ and PMA in comparison to primary human monocytes or monocyte-derived macrophages (MDM). Both stimuli induced changes in cell morphology indicative of differentiation but neither showed differentiation comparable to MDM. In contrast, PMA treatment followed by 5 days resting in culture without PMA (PMAr) increased cytoplasmic to nuclear ratio, increased mitochondrial and lysosomal numbers and altered differentiation-dependent cell surface markers in a pattern similar to MDM. Moreover, PMAr cells showed relative resistance to apoptotic stimuli and maintained levels of the differentiation-dependent anti-apoptotic protein Mcl-1 similar to MDM. PMAr cells retained a high phagocytic capacity for latex beads, and expressed a cytokine profile that resembled MDM in response to TLR ligands, in particular with marked TLR2 responses. Moreover, both MDM and PMAr retained marked plasticity to stimulus-directed polarization. These findings suggest a modified PMA differentiation protocol can enhance macrophage differentiation of THP-1 cells and identify increased numbers of mitochondria and lysosomes, resistance to apoptosis and the potency of TLR2 responses as important discriminators of the level of macrophage differentiation for transformed cells.

## Introduction

Differentiated tissue macrophages arise from monocytes recruited from the blood [1]. Once differentiated, macrophages become long-lived cells and develop specialised functions. Cell numbers are maintained by resistance to constitutive apoptosis [2], by recruitment of further monocytes from the blood and/or replication of local intermediates depending on the prevailing stimulus and anatomical location [3], [4].

Macrophages exhibit marked phenotypic heterogeneity [5]. Functional diversity results from a differentiation programme that is subject to environmental imprinting [6]. Exogenous stimuli such as micro-organisms further modify the selection of phenotype. Although differentiated there is considerable plasticity in the tissue macrophage phenotype; with the current phenotype dependent on the prevailing pattern of stimulation. Major functions of macrophages include maintaining tissue homeostasis and responding to micro-organisms[5]. Macrophages mediate innate immune responses and contribute to adaptive immunity via antigen processing.

Monocytic cell lines of varying degrees of differentiation are frequently used to model macrophage function since primary tissue macrophages cannot be readily expanded *ex vivo*. Isolation requires blood donation or collection from specific tissue by invasive procedures such as bronchoscopy or tissue biopsy [7]. Limited cell numbers represent a barrier to the use of these primary cells in protocols requiring very large numbers of cells. While monocytic cell lines have obvious advantages in terms of ease of acquisition, as compared to primary macrophages, their differentiation state has meant that inferences drawn from these experiments may not always accurately predict the behaviour of differentiated tissue macrophages. To address this, differentiation protocols have been developed treating monocytic cell lines such as U937, HL-60 or THP-1 cells with stimuli such as phorbol-12-myristate-13-acetate (PMA) or 1,25-dihydroxyvitamin D_3_ (VD_3_) [8], [9], [10]. The phenotype of the macrophages in these protocols varies with the differentiation treatment and duration, reflecting differences in gene transcription [11], [12]. The promyelocytic leukaemia cell line HL-60 differentiates towards a monocytic phenotype in response to VD_3_ [13] or a macrophage-like cell in response to phorbol esters, such as PMA [14]. PMA treatment, which activates protein kinase C (PKC), also induces a greater degree of differentiation in THP-1 cells as reflected by increased adherence and expression of surface markers associated with macrophage differentiation [15]. However, it is unclear how the differentiation state of PMA-treated THP-1 cells compares with that of tissue macrophages or monocyte-derived macrophages (MDM), a well recognised model of differentiated tissue macrophages [16].

In the present study we examined differentiation of THP1 cells in response to VD_3_ and PMA stimulation as compared to monocytes and macrophages. Analysis of cell morphology, cell adhesion, expression of surface markers and phagocytic capacity illustrated that while both VD_3_ and PMA stimulation induced macrophage differentiation important differences existed in comparison to MDM. A protocol in which THP1 cells were activated with PMA then rested in culture (PMAr) more closely resembled the phenotype of human MDM.

## Methods

### Cell Culture and Differentiation

Human peripheral blood mononuclear cells (PBMC) were isolated by Ficoll Paque (GE healthcare) density centrifugation from whole blood donated by healthy volunteers. The South Sheffield Research Ethics Committee approved the studies, and subjects gave written, informed consent. Monocytes were enriched from freshly isolated PBMC using MACS Monocyte Isolation Kit II and MACS LS Columns (Miltenyi Biotec), yielding an average 98% purity. To differentiatiate PBMC into monocyte-derived macrophages (MDM) 2×10^6^ PBMC/mL were plated in RPMI 1640 media (Lonza) with 2 mmol/L L-glutamine (Gibco BRL) containing 10% human AB serum (First Link (UK) LTD) in 24-well plates (Costar). After 24 h, non-adherent cells were removed, and adherent cells were cultured in RPMI with 10% heat-treated fetal bovine serum (FBS; Bioclear) in 5% CO_2_ at 37°C to give a final concentration of approximately 2×10^5^ MDM/ml at 14d [17]. The THP1 cell line was obtained from ATCC, and maintained at 2×10^5^ cells/ml in RPMI 1640 medium supplemented with 10% FCS and 2 mmol/L L-glutamine. THP1 cells (2×10^5^/ml) were differentiated using 100 nM Vitamin D_3_ (VD_3_, Sigma-Aldrich) or 200 nM phorbol 12-myristate 13-acetate (PMA, Sigma-Aldrich) for 3d. Differentiation of PMA treated cells was enhanced after the initial 3d stimulus by removing the PMA-containing media then incubating the cells in fresh RPMI 1640 (10% FCS, 1% L-glutamine) for a further 5d (PMAr).

### Flow Cytometry

Flow cytometric measurements were performed using a four colour FACSCalibur (Becton Dickinson). Forward and side scatter light was used to identify cell populations and measure size and granularity of the cells. Auto-fluorescence was recorded by analysing unstained cells in the FL-1 channel (blue laser; excitation 488, emission 530/30). Fc receptors were blocked by incubating 100 µg recombinant human IgG (Sigma-Aldrich) with cells for 15 min at 4°C prior to antibody staining. For detection of cell surface markers 1 µg of monoclonal mouse anti-human antibodies IgG1κ CD14-PE, IgG2aκ TLR2-PE, (eBioscience) and IgG1κ CD206-PE (BD Biosciences) or the relevant isotypes were incubated with samples containing 2×10^5^ cells for 15 min at 4°C. Following incubation samples were washed and resuspended in PBS and 10,000 events recorded. To detect intracellular organelles or intracellular nitric oxide (NO) 2×10^5^ cells were incubated in phenol-red free RPMI 1640 containing 10% FCS and 1% L-glutamine with 1 µM of the nuclear dye Hoescht 33342 (Calbiochem) and either 250 nM MitoTracker Red 580 (Molecular Probes), 500 nM LysoTracker Red DND-99 (Molecular Probes), or with 5 µM of the the pH insensitive fluorescent dye DAF-FM diacetate (Molecular Probes) for 30 min at 37°C [18]. Following incubation all cells were washed in PBS and fluorescence was compared to unstained controls with 10,000 events recorded. All data was analysed using FlowJo software, version 8.8.4 (Tree Star Inc.).

### Confocal Microscopy

Images of whole cell morphology and of mitochondrial or lysosomal staining were acquired, after staining as above, except that 500 nM MitoTracker Red 580 for 30 min at 37°C was used for microscopy. Image capture was performed using a Zeiss LSM 510 confocal microscope and processed by AxioVision 4.7.2 software.

### Latex Bead and Apoptotic Body Phagocytosis

Carboxylate-modified red fluorescent latex beads with a mean diameter of 2 µm (L3030; Sigma-Aldrich) were opsonized in 10% human AB serum with RPMI 1640 for 30 min at 37°C. To account for extracellular binding, phagocytosis was inhibited by adding 5 µM Cytochalasin D (Sigma-Aldrich) to control samples for 30 min (37°C, 5% CO_2_) prior to infection to block F-actin dependent phagocytosis. The median fluorescence intensity of these samples was subtracted from samples exposed to beads in the absence of Cytochalsin D [17]. Opsonized beads were incubated with cells at a multiplicity of infection of 10 for 4 h. Cells were washed twice in PBS and measured by flow cytometry. Flow cytometry was performed to estimate the number of latex beads ingested per cell with each peak assumed to equal an additional bead. Results were repeated with fluorescence microscopy confirming approximately the same number of beads per cell. In some experiments apoptotic neutrophils were used in place of beads. Human peripheral blood neutrophils were cultured for 20 h, resulting in 70–80% apoptotic cells, and less than 5% necrotic cells by trypan blue exclusion. Apoptotic neutrophils were fed to THP-1, monocytes and macrophages for 90 min Phagocytosis was assessed by MPO staining and phagocytic index calculated by multiplying the percentage of cells that had engulfed neutrophils by the average number of neutrophils per cell [19].

### Cytokine Production

THP1/Monocyte/MDM cultures were stimulated with 10 ng/ml ultra-pure LPS (Alexis Corporation) or 100 ng/ml Pam3CSK4 (InvivoGen). Supernatants were harvested after 20 h, and stored at −80°C until analysis. TNF-α, IL-1β, IL-6 and IL-8 levels were measured by ELISA (R & D Systems) according to manufacturer's instructions. Limits of detection were 1.6 pg/ml and 4.8 pg/ml for IL-1β and TNF-α, respectively.

### Apoptosis Induction

Apoptosis was induced by UV irradiation (1500 µJ/cm^2^, Stratalinker 1800, Stratagene) followed by 4 h culture, or 1 µM staurosporine (Calbiochem) for 16 h. Cells were subsequently fixed with methanol, and nuclear morphology was examined by DAPI as previously described [17]. Separate cultures were treated with 1 µM staurosporine for 16 h for western blot analysis of Mcl-1 and detection of caspase 3 or 7 activation using Caspase-Glo® 3/7 Assay (Promega).

### Western Blot Analysis of Mcl-1

Cells were lysed in SDS buffer (20 mM Tris-HCl pH 7.4, 150 mM NaCl, 5 mM EDTA, 5 mM EGTA and 1% SDS) containing complete protease inhibitor cocktail (Roche). Protein concentration was determined by the Bradford protein assay and gels loaded with equal amounts of protein per lane. Electrophoretic separation (15–40 µg protein/lane) was carried out on 15% polyacrylamide gels, and subsequently transferred to Immobilon-P membrane (Millipore). Membranes were blocked in PBST/5% non fat dry milk powder and incubated with primary and secondary antibodies as described previously [20]. The antibodies used in this study were: anti-human Mcl-1 (SC-19) and Bax (N-20) (Santa Cruz Biotechnology), and actin (A2086, Sigma-Aldrich). Protein was detected using HRP-conjugated goat anti-rabbit immunoglobulins (Dako), and enhanced chemiluminescence (Amersham Pharmacia).

### Measurement of Macrophage Polarization

PMAr or MDM were cultured in the absence or presence of heat-killed type 2 *S. pneumoniae* (D39) at a multiplicity of infection of 10 and expression of macrophage mannose receptor (CD206) was detected by flow cytometry after 72 h culture.

### Statistical Analysis

All data was recorded as mean ± standard error of the mean (se) unless otherwise stated. Statistical testing was performed using Prism® 5.02 software (GraphPad Software Inc.) with the statistical tests as shown in the figure legends. Significance was defined as p<0.05.

## Results

### Morphological Characteristics of THP-1 Cells Following Differentiation

**Figure 1:**
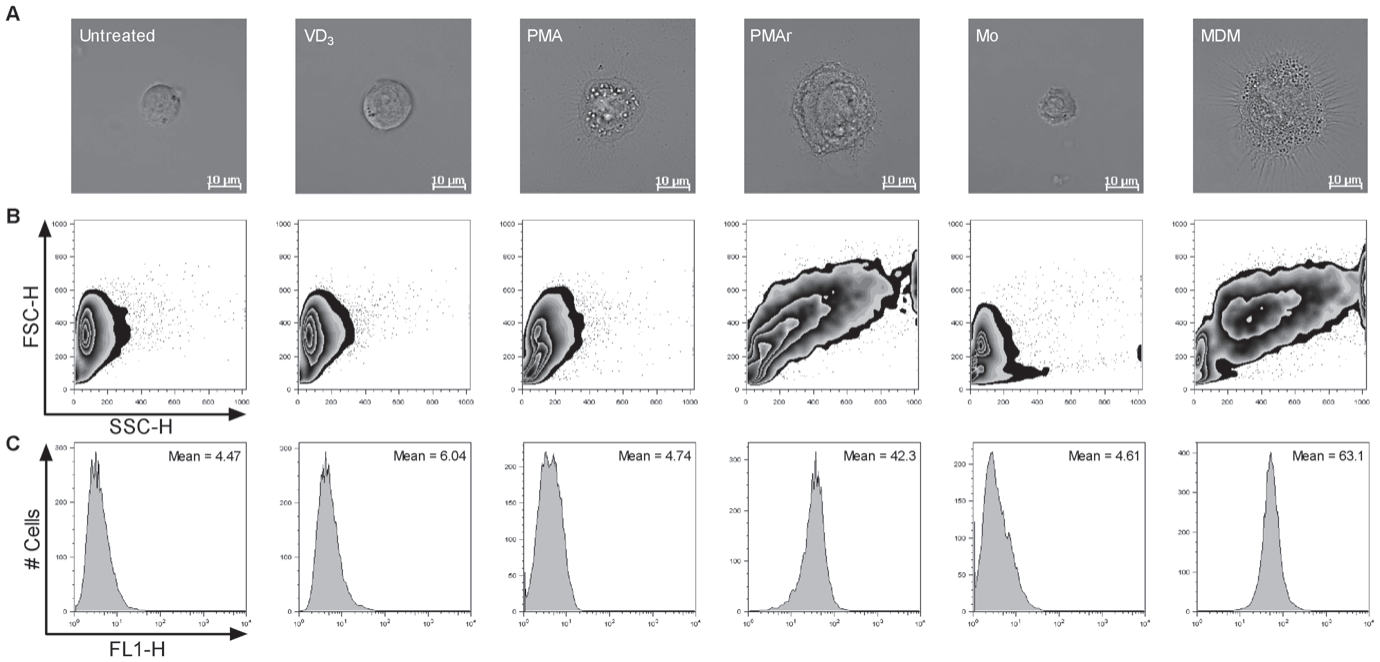
Morphological changes of macrophages with differentiation. Representative differential interface contrast image (A), forward light scatter and side light scatter plot (B) and histograms of autofluorescence, with the mean fluorescence intensity shown in the upper right hand corner, (C) of THP-1 cells untreated, treated with Vitamin D3 (VD_3_), PMA or treated with PMA and subsequent resting (PMAr), and of monocytes (Mo) or monocyte-derived macrophages (MDM). Data is representative of at least three independent experiments.

Macrophage differentiation is associated with a reduction in the nucleocytoplasmic ratio due to an increase in cytoplasmic volume [21]. As anticipated human mononocyte-derived macrophages (MDM) increased their cytoplasmic volume as compared to monocytes (Figure 1A). VD_3_ and PMA treatment also increased the cytoplasmic volume in THP-1 cells relative to monocytes. PMA treatment enhanced the adherence of the THP-1 cells relative to untreated cells but did not induce the firm adherence of MDM (data not shown). If cells were treated with PMA and then rested by culture for a further 5 days in the absence of PMA (PMAr) they showed a much greater increase in cytoplasmic volume and more closely resembled MDM with much firmer adherence.

Another feature of macrophage differentiation is enhanced granularity, as demonstrated by increase in side scatter (SSC) on flow cytometry [22], [23]. This results from an increase in the number of certain membrane bound organelles [21], [24]. A further distinguishing feature of macrophage differentiation is autofluorescence, which is marked in differentiated macrophages such as alveolar macrophages but low in other myeloid cell types such as monocytes and dendritic cells [25]. MDM have increased SSC and increased autofluorescence on flow cytometry but THP-1 cells treated with VD_3_ or PMA had much lower SSC and autofluorescence than MDM (Figure 1B–C). In contrast PMAr cells increased their granularity and autofluorescence to a level comparable to that of MDM. Similar results were obtained regardless of whether the initial period of PMA stimulation included VD_3_ treatment or not, or if the rest period of culture following PMA stimulation lasted for 3d or up to 7d. However, the phenotype was not replicated by initial treatment with VD_3_ in the absence of PMA, followed by a further 3d of culture without PMA or VD_3_ stimulation (data not shown). Since VD_3_ stimulation or prolonged periods of rest neither enhanced nor inhibited the degree of differentiation, further experiments were carried out using the 3d treament-5d resting protocol with PMA alone. Since VD_3_ or PMA stimulation without resting was similar in initial phenotype, further comparisons included just the VD_3_ stimulation without resting treatment.

### PMA Stimulation Followed by Resting Increases the Concentration of Lysosomes and Mitochondria in THP-1 Cells

**Figure 2:**
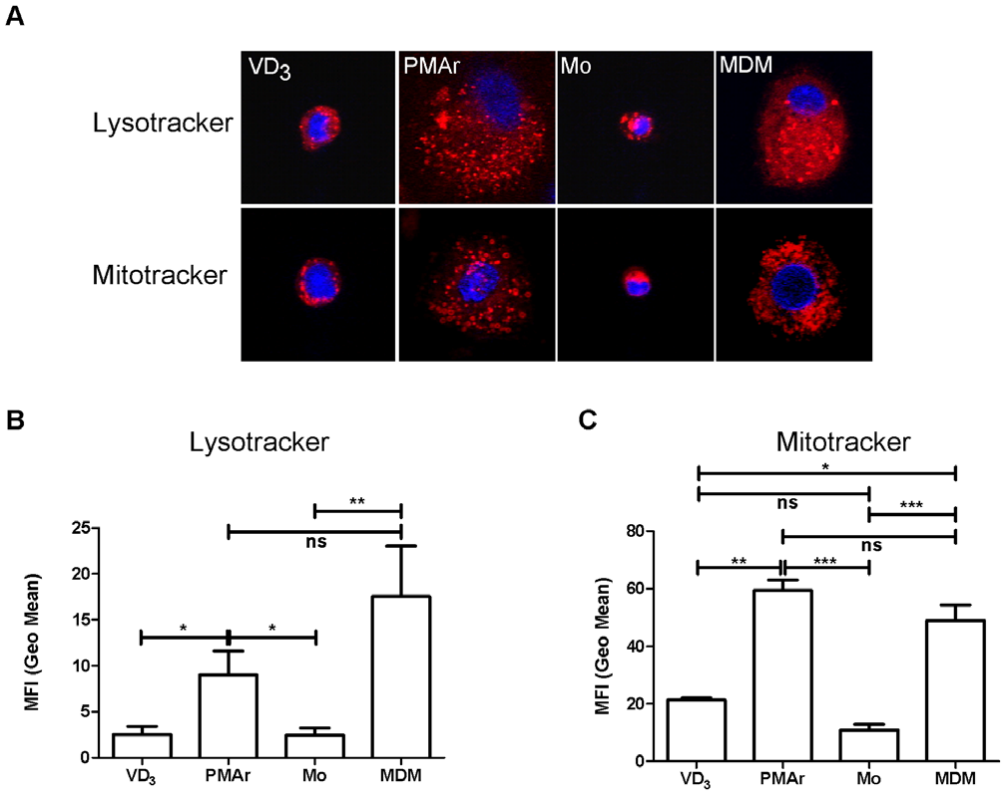
Increasing numbers of lysosomes and mitochondria with macrophage differentiation. Representative confocal images after LysoTracker staining for lysosomes and MitoTracker staining for mitochondria in THP-1 cells treated with Vitamin D_3_ (VD_3_) or PMA treated and rested (PMAr) and monocytes (Mo) or monocyte-derived macrophages (MDM), (A). Median fluorescence intensity (MFI) after staining with LysoTracker (B) or Mitotracker (C) and analysis by flow cytometry, n = 5, ns p>0.05, * p<0.05, ** p<0.01, *** p<0.001, ANOVA.

To confirm the increased SSC appearances on flow cytometry reflected increased numbers of cellular organelles in the cytoplasm, we next stained for two organelles whose cytoplasmic number contributes to macrophage's increased light scattering, namely mitochondria and lysosomes [24], [26] and which accumulate with macrophage differentiation [27]. As depicted in Figure 2, the PMAr had a greater intensity of lysosomal staining than monocytes or VD_3_ treated THP-1 cells (Figure 2A–B). The pattern and intensity of mitochondrial staining in PMAr with broad distribution throughout the cytoplasm was more similar between the PMAr cells and MDM (Figure 2A and 2C). A combined approach of microscopy for localisation and flow cytometry for overall signal intensity confirmed that the PMAr THP-1 cells were most similar to MDM.

### Surface Markers of THP-1 Cells during Differentiation Protocols

**Figure 3:**
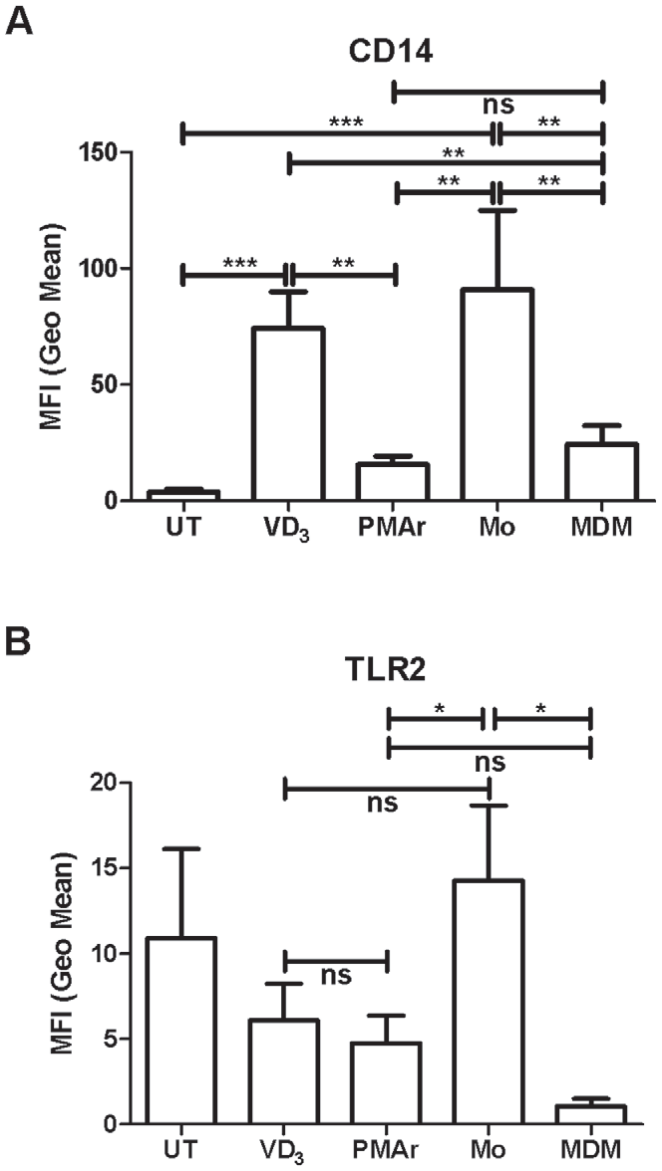
Cell surface marker expression with macrophage differentiation. Graph of median fluorescent intensity (MFI) of CD14 (A) and TLR2 (B) expressed on the cell surface of THP-1 cells untreated (UT), treated with Vitamin D_3_ (VD_3_) or PMA treatment with resting (PMAr) and monocytes (Mo) or monocyte-derived macrophages (MDM), n = 3–5, ns p>0.05, * p<0.05, **, p<0.01, *** p<0.001, Mann-Whitney U test.

CD14 is a monocyte marker that is downregulated during differentiation [28]. VD_3_ treated cells and monocytes have greater CD14 surface expression in comparison to PMAr and MDM (Figure 3A). TLR2 is another surface marker that is downregulated with macrophage differentiation [29]. There was no statistical difference between MDM and PMAr THP-1 cells. THP-1 differentiation protocols resulted in cells that expressed only low levels of surface TLR2 comparable to MDM but unlike the higher levels observed with monocytes (Figure 3B), but once again PMAr THP-1 cells and MDM were not statistically different. These data support the differentiation of PMAr and VD_3_ treated cells away from a monocyte phenotype but suggest this has occurred to a greater extent for PMAr cells.

### Resistance to Apoptosis with THP-1 Differentiation

**Figure 4:**
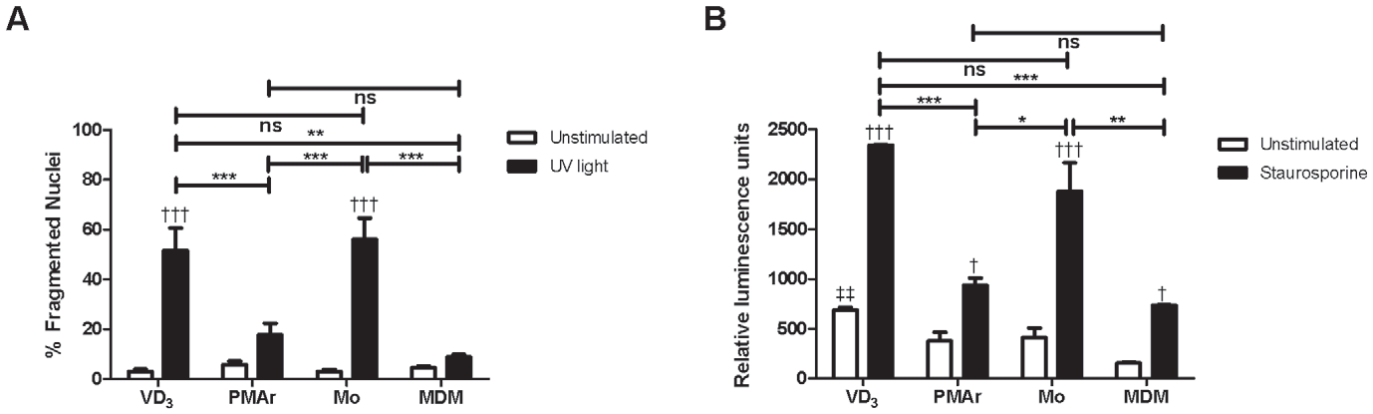
Apoptosis susceptibility is decreased with macrophage differentiation. Graph of percent fragmented nuclei by fluorescence microscopy 4 hr after UV exposure stained with DAPI (A) and relative caspase 3/7 activation 16 hr after staurosporine treatment (B) of THP-1 cells treated with Vitamin D_3_ (VD_3_) or PMA and rested (PMAr) and monocytes (Mo) or monocyte-derived macrophages (MDM), n = 3–6, † p<0.05, ††† p<0.001, two-way ANOVA with Bonferroni post tests; ns p>0.05, * p<0.05, ** p<0.01, *** p<0.001, ‡‡ p<0.01 vs. MDM, ANOVA with Bonferroni post tests.

Monocytes are susceptible to constitutive apoptosis and apoptosis is readily induced with a variety of stimuli [30]. In contrast MDM and tissue macrophages are less susceptible to stimuli that induce monocyte apoptosis such as cytokine withdrawal, in keeping with their status as long-lived tissue cells [31]. At the doses studied UV irradiation induced apoptosis at varying levels. As anticipated MDM were relatively resistant while monocytes were susceptible (Figure 4A). PMAr cells also demonstrated lower levels of apoptosis, broadly comparable to MDMs and significantly less than VD_3_ treated cells or monocytes. These findings were confirmed by showing that different macrophage cell types also showed differential sensitivity to a second pro-apoptotic stimulus, staurosporine, when analysed by morphology (data not shown) or caspase 3/7 activation (Figure 4B). All cell types showed an increase in apoptosis with treatment but there was significantly less apoptosis in MDM and PMAr THP-1 cells as compared to VD_3_ treated THP-1 or monocytes.

**Figure 5:**
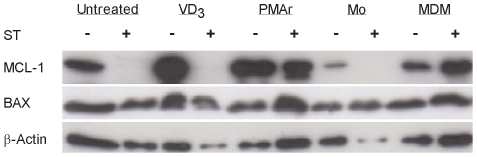
Mcl-1 upregulation with macrophage differentiation. Representative Western blot probed for Mcl-1, Bax and β-actin from THP-1 cells untreated, treated with Vitamin D_3_ (VD_3_) or PMA and rested (PMAr) and monocytes (Mo) or monocyte-derived macrophages (MDM) following culture in the absence (−) or presence(+) of staurosporine (ST) for 16 h. Data is representative of three independent experiments.

Mcl-1 is a critical regulator of macrophage resistance to apoptosis and its expression is differentiation dependent [30]. Constitutive expression was lower in monocytes than other cells (Figure 5). In response to staurosporine, PMAr THP-1 and MDM maintained Mcl-1 expression but VD_3_ treated THP-1, like monocytes, down-regulated Mcl-1. Apoptosis levels assessed by changes in nuclear morphology were high in monocytes (57.5%±19%, n = 6), but lower in macrophages (16.8%±1.7%, n = 6). As shown, the downregulation was not part of a general downregulation of Bcl-2 proteins since Bax, another family member less susceptible to dynamic changes, showed constant levels in all cells while actin, which is degraded in late stage apoptosis, showed relative preservation in comparison to Mcl-1.

### Capacity of Differentiated THP-1 to Phagocytose Opsonized Particles

**Figure 6:**
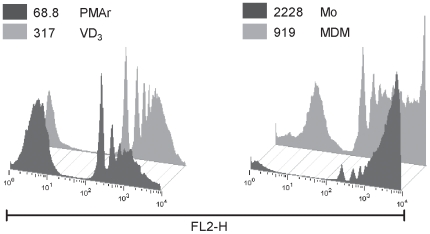
Phagocytosis of latex beads in macrophages. THP-1 cells treated with Vitamin D_3_ (VD_3_) or PMA and rested (PMAr) and monocytes (Mo) or monocyte-derived macrophages (MDM) were challenged with latex beads and phagocytosis measured by flow cytometry. Histograms depict representative data from four independent experiments. MFI for the illustrated experiments were as follows: VD_3_ (317), PMAr (68.8), monocytes (2228) and MDM (919).

To further characterize the responses of PMAr cells we investigated phagocytic capacity. In general, differentiated macrophages retain a significant capacity to phagocytose opsonized particles. As shown in Figure 6, all cell types tested phagocytosed opsonized beads. The percentage of ingesting cells was broadly similar between different cell types (Table 1). MDM showed a non-significant trend towards less phagocytosis of beads. The PMAr THP-1 cells ingested similar numbers of beads per cells to the MDM and significantly fewer beads per cell when compared to the monocytes. Similar results were obtained when apoptotic neutrophils were fed to macrophages, with a comparable phagocytic index in PMAr THP-1 cells and MDM (See Table 1).

**Table 1:** Phagocytosis of latex beads and apoptotic neutrophils in different phenotypes of monocyte/macrophages.

Cell	Phagocytosing cells (%) (Flow cytometry)	Number of beads per phagocytosing cell (Flow cytometry)	Number of beads per phagocytosing cell (Microscopy)	Phagocytic index (Apoptotic neutrophils)
VD_3_	43.2±13.2	3.5±0.8	2.7±0.3**	11.6±5.2
PMAr	66.1±5.5	4.8±1.1*	8.4±0.8*	14.6±2.8
Mo	78.6±8.8	17.2±5.6	21.8±8.3	55.9±23.5
MDM	70.5±11.2	7.6±4.1	8.1±0.5*	38.8±28

THP-1 cells treated with Vitamin D_3_ (VD_3_), PMA and rested (PMAr), monocytes (Mo) or monocyte-derived macrophages (MDM) were challenged with latex beads and apoptotic neutrophils. Phagocytosis was measured by flow cytometry and fluorescence microscopy (mean ± std. deviation), n = 3–4, * p<0.05, ** p<0.01 *vs*. Mo, ANOVA with Bonferroni post tests.

### Cytokine Profiles of TLR Stimulated THP-1 Cells

**Figure 7:**
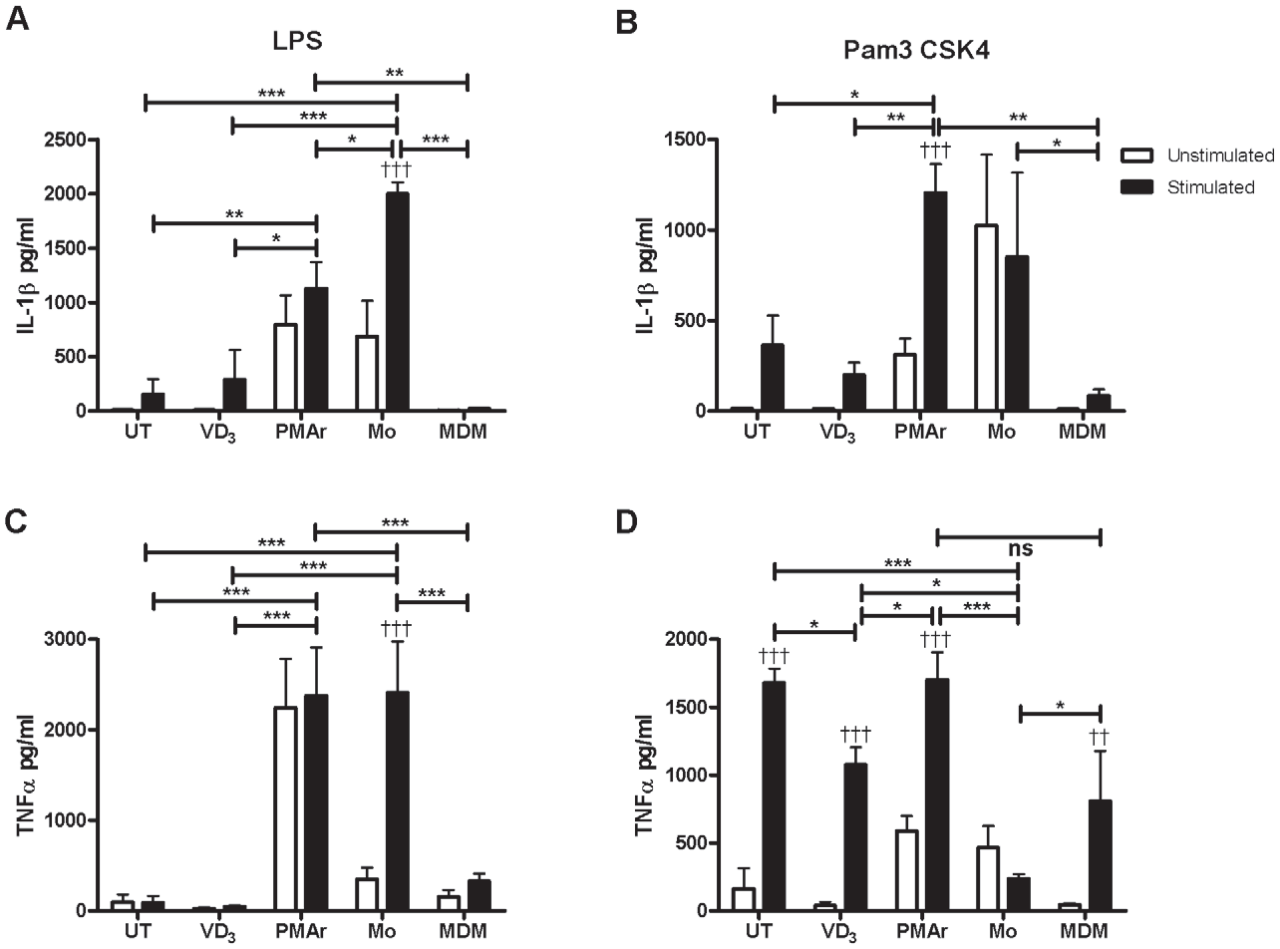
Differential cytokine expression with macrophage differentiation. THP-1 cells treated with Vitamin D_3_ (VD_3_) or PMA and rested (PMAr) and monocytes (Mo) or monocyte-derived macrophages (MDM) were stimulated with LPS or Pam3CSK4. IL-1β (A and B) and TNF-α (C and D) were measured from supernatants, n = 5–9, † p<0.05, †† p<0.01, ††† p<0.001, two-way ANOVA with Bonferroni post tests; * p<0.05, ** p<0.01, *** p<0.001, ANOVA with Bonferroni post tests.

Important differences in macrophage expression of certain well characterised cytokines exist. IL-1β is markedly induced following TLR2 or TLR4 ligation of monocytes while this response is downregulated in many differentiated tissue macrophages such as alveolar macrophages [32]. LPS only stimulated significant induction of IL-1β and TNF-α in monocytes. Despite variable constitutive levels none of the other cell types had significant cytokine induction with LPS (Figure 7A and C). IL-6 and IL-8 levels were also more markedly induced in monocytes following LPS treatment (data not shown).

In contrast, the TLR2 ligand Pam3CSK4 produced a more marked induction of cytokine expression from the more differentiated macrophages, despite variable absolute levels, and there was no induction of the two cytokines in monocytes (Figure 7B and D). Although IL-1β levels for monocytes after TLR2 stimulation remained high they were largely constitutive and TNFα levels were statistically higher for all cells in comparison to monocytes in intra-group comparisons. Thus monocytes were more responsive to LPS while MDM and the THP-1 cells undergoing differentiation were more responsive to the TLR2 agonist with similar patterns of both IL-1β and TNFα production following stimulation.

### Induction of NO in Macrophage Populations

**Figure 8:**
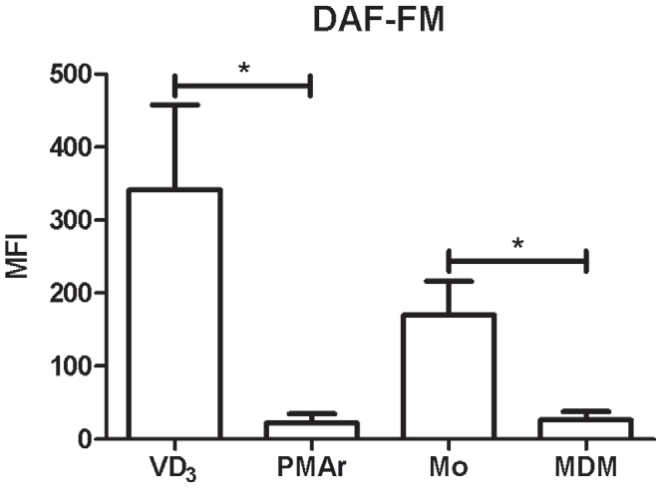
Low NO production in differentiated macrophages. THP-1 cells treated with Vitamin D_3_ (VD_3_) or PMA and rested (PMAr) and monocytes (Mo) or monocyte-derived macrophages (MDM) were stimulated with LPS and NO detected by DAF-FM staining and flow cytometry, n = 4, * p<0.05, Mann-Whitney U test.

Previous work has emphasised that although induction of NO may be less marked in human as compared to rodent macrophages, a variety of conditions can stimulate NO production via nitric oxide synthase 2 (NOS2) in human macrophages [33]. LPS alone can stimulate NO production in human monocytes [34], but when added alone, i.e. without additional cytokine stimulation, is insufficient to induce NOS2 and therefore NO in MDM [35]. As anticipated therefore we found low but detectable levels of NO production in monocytes but no detectable levels in MDM (Figure 8). Of note VD_3_ treated THP-1 behaved like monocytes and PMAr THP-1 cells like MDM. This is in keeping with the greater induction of cytokines from monocytes by LPS which may act synergistically with LPS to stimulate NO expression [33].

### Activation Status of PMA Rested THP-1 Cells in Comparison to MDM

**Figure 9:**
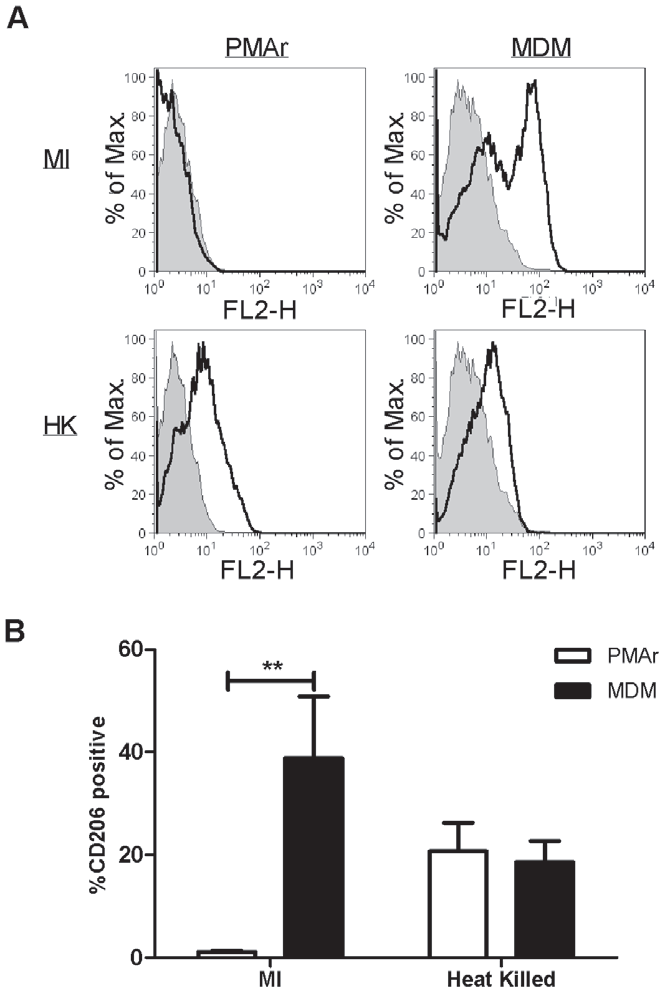
Differentiated Macrophages retain plasticity of polarization. THP-1 cells treated with PMA and rested (PMAr) and monocyte-derived macrophages (MDM) were cultured in the absence (MI) or presence of heat killed pneumococci (HK) and expression of the macrophage mannose receptor (CD206) detected by flow cytometry. Data shows representative histogram overlays of isotype (filled histogram) and CD206 stained cells (transparent histogram) (A) with the percentage CD206 positive cells (B), n = 3, ** p<0.01, two-way ANOVA with Bonferroni post tests.

PMAr THP-1, although similar to MDM in morphology and LPS hyporesponsiveness, demonstrated greater levels of constitutive and inducible IL-1β and TNF-α production following TLR2 or TLR4 stimulation. These results suggested that PMAr THP-1 might have a different activation state to MDM. Macrophages have been regarded as either classically or alternatively activated, although the latter group may contain several different macrophage sub-types [5], [36]. To explore this we determined if the macrophage mannose receptor, CD206, a marker of alternatively activated macrophages was differentially expressed between MDM and PMAr THP-1 cells [37]. THP-1 cells express CD206 in certain differentiation protocols [38]. As shown in Figure 9, CD206 was detected in MDM in approximately half of the MDM population but not the PMAr THP-1 cells. Macrophages can sense the microenvironment they are exposed to and adapt polarity [39]; this plasticity is a key feature of macrophages. Despite the apparent differences in phenotype prior to exposure to heat killed bacteria the percentage of CD206 cells became similar for the two cell types following bacterial stimulation, with the majority of cells being CD206 null, in keeping with a classically-activated phenotype. This suggests that, despite initial differences in apparent phenotype, the polarization became similar following microbial stimulation; both cell types showed the ability to adapt their phenotype.

## Discussion

Macrophage heterogeneity is influenced by differentiation state, with marked differences between monocytes and macrophages [1], [40]. Commonly used protocols induce macrophage differentiation in monocytic cell lines but derivation towards a highly differentiated macrophage phenotype has not been a major focus of prior studies. In this study we have identified a number of macrophage characteristics associated with differentiation which vary in THP-1 cells treated with different protocols. These characteristics include the expansion of the cytoplasm and of cytoplasmic organelles (such as mitochondria and lysosomes), the capacity to upregulate the anti-apoptotic protein Mcl-1 and resist apoptotic stimuli and the responsiveness to TLR2 or 4 ligands. We demonstrate that a protocol of treating THP-1 cells with PMA followed by a period of further culture without PMA drives cells towards a differentiated macrophage phenotype that more closely resembles MDM than does differentiation with PMA or VD_3_ at doses commonly used in the literature [8]. Since MDM represent a good surrogate for tissue macrophages, such as alveolar macrophages, this suggests the PMAr cells may also represent a useful model with which to study tissue macrophages [16].

The phagocytic capacity of macrophages is central to their function. This principally involves the clearance of cellular debris during physiological homeostasis but also the removal of exogenous particles, including micro-organisms [5]. Ingested particles are contained in phagosomes which fuse with endosomes and lysosomes to mature into organelles capable of digesting the phagocytosed particles [41]. Lysosomes are central effectors of macrophage function, containing a range of proteases capable of degrading ingested particles [42]. Degradative capacity is linked to the maintenance of an acidic milieu in the lysosome, an energy dependent process requiring the vacuolar-type proton ATPase (V-ATPase) and other hydrogen pumps, which are required for activation of the proteases [43], [44]. Commensurate with the increased degradative capacity inherent in the greater numbers of lysosomes is a requirement for greater energy generation via mitochondria. In keeping with prior publications showing an increased number of lysosomes and mitochondria in differentiated macrophages, we identified increased numbers of mitochondria in all of the macrophage phenotypes as compared to monocytes [27], [45]. However the increase in lysosomes was most marked for PMAr THP-1 cells in which levels were comparable to those in MDM. In view of the greater mitochondrial numbers and development of larger lysosomal structures in macrophages, as opposed to the primary granules of monocytes, these differences are likely to have significant functional consequences in terms of the kinetics with which particles are internalized and degraded [45], [46].

Cytokine responses induced in monocytes as compared to macrophages vary depending on the stimulus and the type of differentiated macrophage studied. LPS induction of TNF-α is often observed to be greater in monocytes than macrophages due to transcriptional or translational upregulation [47], [48], although some studies have demonstrated increased production in macrophages [49]. Differences likely reflect alternative differentiation protocols, variability in the LPS purity and the impact of LPS tolerance [50], [51]. Enhanced IL-1β secretion by monocytes following TLR stimulation is also well recognised [47]. This occurs since monocytes have constitutive activation of caspase-1, required for IL-1β processing and secretion, while macrophages require a second stimulus to activate caspase-1 [52]. We deliberately only studied cells challenged without added ATP treatment since this has been the method most often employed in the literature, but further comparisons with ATP co-stimulation would also be informative. Although we also observed these patterns, we also noted that the more differentiated macrophages had higher inducible responses to the TLR2 ligand Pam3CSK4. Interestingly, alveolar macrophages have been demonstrated to have enhanced TNF-α responses to mycobacteria, in comparison to monocytes, a finding which may also link to the enhanced TLR2 responses we observed [53]. The pattern of cytokine production was broadly supported by analysis of NO production. The monocytes produced NO following LPS stimulation while the MDM and PMAr THP-1 cells showed no production of NO. The lack of NO production is not due to an inability of MDM to produce NO since, for certain stimuli such as *Streptococcus pneumoniae*, MDM produce NO [18]. Since NO production from human macrophages in response to LPS requires synergistic stimulation with cytokines [33], it is likely that the greater expression of TNF-α and related cytokines in LPS-stimulated monocytes was an important factor in driving NO production.

Differences in the initial polarization of the MDM and PMAr THP-1 cells were noted. Characterization involving a number of features that discriminate between classically activated (M1) and alternatively activated (M2) macrophage phenotypes was undertaken [5]. The demonstration of significant CD206 expression, absent NO production and lower levels of LPS responsiveness were in keeping with significant M2 polarization within the MDM population [5]. In contrast the PMAr THP-1 cells, although having other features which suggested similar differentiation to MDM, had no detectable CD206 expression when resting and higher levels of constitutive and TLR2 induced TNF-α and IL-1β. These features suggested a greater degree of classical activation. However features of polarization are likely to represent a continuum within a population of cultured cells and to be highly adaptable to prevailing stimuli [39]. The MDM challenged with heat killed bacteria showed greater features of classical activation. Of interest, the PMAr THP-1 cells, although still showing a predominant classically activated phenotype, shifted their level of CD206 expression and became much more similar in overall phenotype to the MDM. This data suggested that, although differences in baseline phenotype may exist, both cell types retain the functional capacity to adapt to the prevailing stimulus and to shift their polarization to a common stimulus-directed phenotype. In this regard the findings fit well with the view that functional competence is more closely related to the prevailing stimuli than the lineage from which the mononuclear phagocyte originates. Such arguments have for example been used to suggest that antigen presentation is a function shared more broadly by mononuclear phagocytes than may have been appreciated previously and in the same way key innate host defense functions may be adaptable [54].

A defining feature of differentiated tissue macrophages, in comparison to monocytes, is their resistance to constitutive apoptosis resulting from serum withdrawal or culture in the absence of pro-survival cytokines [31]. Monocytes are highly susceptible to apoptotic stimuli and apoptosis represents the fate of monocytes that migrate into tissue and do not encounter pro-survival stimuli such as LPS, growth factors and cytokines such as TNF-α and IL-1β [55]. Macrophage resistance is achieved by upregulation of anti-apoptotic factors including Fas-associated death domain-like interleukin 1beta-converting enzyme-inhibitory protein (Flip) and phosphatidylinositol 3-kinase dependent increases in Mcl-1expression [30], [56]. Unlike MDM, standard differentiation protocols involving VD_3_ produced macrophages that retained significant susceptibility to apoptosis and which failed to maintain Mcl-1 expression in the presence of pro-apoptotic stimuli such as staurosporine. The PMAr cells were resistant to these apoptotic stimuli and did not downregulate Mcl-1 with the doses of staurosporine studied. Another Bcl-2 member Bax did not show differential regulation. Differing activation states can modify macrophage susceptibility to apoptosis [31], but in view of the other features suggesting differences in differentiation we observed it seems that differentiation state, rather than activation state, was the major factor contributing to the altered susceptibility to apoptosis we observed. This was enforced by the observation that the two most differentiated macrophage phenotypes, MDM and PMAr, showed differing states of activation but similar resistance to apoptosis.

The implications of these findings should be carefully considered when designing models with which to investigate macrophage function. While standard differentiation protocols may provide informative models for the investigation of many macrophage responses they will have important differences in comparison to tissue macrophages. The altered volume of the lysosomal compartment will potentially modify their degradative capacity. The differences in mitochondrial number may impact their capacity to match critical functions to ATP generation with metabolic consequences. Differences in responses to particular TLR ligands will have consequences for investigations of particular pathogens which may preferentially utilise specific patterns of TLR [57]. Altered regulation and susceptibility to apoptosis will not only have consequences to the investigation of this critical biological function but will have more widespread consequences for the investigation of macrophage function, since regulation by apoptosis is an important control point over function [20], [31].

Our findings apply in particular to differentiated tissue macrophages such as alveolar macrophages which have similar attributes to the MDM we studied [16]. They do not take account of specific differences that may arise because of unique environmental factors which stimulate the development of highly specialised tissue macrophages, for example microglia or osteoclasts. For such specialized sub-sets of tissue macrophages different differentiation protocols are likely to be required to hone particular macrophage characteristics. The functional consequences of our observations require further investigation. Nevertheless a modified differentiation protocol involving PMA treatment followed by culture without PMA or VD_3_ generates macrophage with characteristics shared by MDM and lacking in cells treated with other differentiation protocols. These cells will provide an additional tool with which to further investigate the biology of tissue macrophages.
